# Recent Advances in the Diagnosis and Treatment of Brain Tumors

**DOI:** 10.3390/brainsci14030224

**Published:** 2024-02-28

**Authors:** Alberto Delaidelli, Alessandro Moiraghi

**Affiliations:** 1 Department of Molecular Oncology, British Columbia Cancer Research Centre, Vancouver, BC V5Z 1L3, Canada; 2 Department of Pathology and Laboratory Medicine, University of British Columbia, Vancouver, BC V6T 1Z4, Canada; 3 Department of Neurosurgery, GHU Paris Psychiatrie et Neurosciences, Hôpital Sainte-Anne, 75014 Paris, France; alessandro.moiraghi@sfits.ch; 4 Institute of Psychiatry and Neuroscience of Paris (IPNP), Université Paris Cité, INSERM U1266, 75014 Paris, France

Brain tumors represent some of the most aggressive malignancies. The latest WHO Classification of Tumors of the Central Nervous System recognizes more than 100 primary brain tumors, highlighting the diagnostic challenges for these tumor entities [1]. Recent advances in neuroimaging and intra-operative techniques allow for the safer diagnosis of brain tumors [2,3], more extended resection and better functional outcomes, especially when they are seated in eloquent areas of the brain [4,5,6,7,8,9,10,11]. However, surgery, even along with current chemoradiotherapy, is often non-curative for the most aggressive tumors such as high-grade gliomas, medulloblastoma, ATRT and many others [12,13]. As a result, many brain tumor survivors experience serious side effects and long-term sequelae, such as permanent neurological deficits, seizures, and learning disabilities [5,14,15,16]. To face these challenges, new promising targeted therapies [17,18,19,20], advanced diagnostic modalities and novel outcome predictors [21,22,23,24,25,26] are rapidly emerging in the current literature. Some of the emerging surgical techniques currently under investigation include focused ultrasound for both tumor ablation and blood–brain barrier disruption to enhance drug delivery [27,28,29,30,31,32], magnetic fields to control tumor progression [33], intra-operative fluorescence and phototherapy to limit malignant cells infiltration [34,35,36], as well as laser tumor ablation [37]. In a recent Special Issue of *Brain Sciences*, entitled “Advances in Diagnosis and Treatment of Brain Tumors”, we curated a collection of research articles focusing on these emerging topics.

Among the review articles published, Martin et al. provided an update on the implications of the 2021 WHO Classification of diffuse adult gliomas for neuropathology diagnosis, including the integration of molecular tests for the diagnostic workup of these malignancies. Similarly, Janelli et al. provided an update on the state-of-the-art therapies for papillary craniopharyngioma, reporting an emerging role of the BRAF/MEK inhibitor therapy not only in the adjuvant setting, but also as a first-line treatment (a neo-adjuvant approach) due to its efficacy and rapidity of action. Additional informative review articles provided an overview on the most commonly utilized photosensitizers for photodynamic therapies and endoscopic approaches for middle-third falcine meningioma. These approaches may allow for a better visualization of the surgical corridor with less visual blindness areas, minimal brain retraction for the preservation of eloquent cortex and bridging veins, and early devascularization without removal of the falcine venous channels.

The research articles published included the description of novel biomarkers for glioblastoma, such as CXCR4, whose mRNA and protein expression represent independent prognostic factors and positively correlates with tumor inflammatory markers. Novel diagnostic strategies for glioma were proposed by Zhou et al. The authors detected the circular RNA hsa_circ_0004214 in glioma tissue, reporting that its expression level represents a promising diagnostic marker. Additional molecular biology research by Chen et al. showed differential spatial distribution of brain metastases in patients with non-small cell lung cancer according to their mutation status. From a surgical standpoint, Vandenbulcke et al. reported that sacrifice of an involved nerve root in foraminal nerve sheath tumors to achieve gross total resection seems to represent a reasonable approach, allowing tumor resection without severe or debilitating long-term dysfunction. Baro et al. successfully utilized functional diffusor tensor imaging (DTI) tractography based on navigated transcranial magnetic stimulation (nTMS) motor mapping for pediatric diencephalic tumors, obtaining valuable information about the lesion and the surrounding eloquent areas. This enabled specific and more accurate surgical planning, helping surgical decision making to improve the extent of resection while preserving neurological function. Finally, a cases series by Shao et al. indicated that endoscopic trans-sphenoidal surgery with a layered peel strategy provided a significant remission rate, low complication rate, and no recurrence in patients suffering from Cushing disease.

Several interesting case reports were also published. For pediatric tumors, a primary intracranial sarcoma DICER1-mutant was described by Kosteniuk et al. In addition, a case of ATRT treated with two-staged surgery and chemotherapy was reported by Paun et al., proposing this multistage resection and oncological treatment to reduce surgical mortality and morbidity, aiming to thus guarantee a good quality of life. Mastrantuoni et al. reported the rare case of a 39-year-old patient with a paraspinal muscle metastasis from a myxopapillary ependymoma. Sun et al. presented the first report describing meningiomatosis combined with calcifying pseudoneoplasm of the neuraxis. Finally, a 16-year follow-up of a 46-year-old woman affected by hemangiopericytoma of the cavernous sinus treated with adjuvant radiotherapy was reported by Detti et al.

While far from being comprehensive, this collection of articles fills some of the knowledge gaps around the clinical and research challenges currently faced in the field of brain cancer. The continually evolving field of brain tumor classification is expected to witness further integration of molecular techniques over the next few years. These include the extension of DNA methylation classifiers to additional tumor types, as well as the utilization of emerging tools for tumor classification and outcome prediction, such as proteomics, microRNA or circular RNA biomarkers. Novel targeted therapies are currently being investigated in preclinical models and early phase clinical trials. Targeted inhibitors, drug-conjugated antibodies and chimeric antigen receptor T cells represent some of the new exciting therapeutic developments promising to solidify their role in the clinical approach to brain tumors. The use of advanced surgical techniques including focused ultrasound, intra-operative fluorescence and phototherapy, and functional DTI tractography based on nTMS will continue to grow worldwide. Overall, the integration of molecular, bioengineering, and surgical technologies is leading the scientific community towards a more refined diagnosis of brain tumors as well as more efficacious and personalized interventions in the near future.
